# Development of Polyhydroxybutyrate-Based Packaging Films and Methods to Their Ultrasonic Welding

**DOI:** 10.3390/ma16206617

**Published:** 2023-10-10

**Authors:** Viktoriia Talaniuk, Marcin Godzierz, Alina Vashchuk, Maksym Iurhenko, Paweł Chaber, Wanda Sikorska, Anastasiia Kobyliukh, Valeriy Demchenko, Sergiy Rogalsky, Urszula Szeluga, Grażyna Adamus

**Affiliations:** 1 Centre of Polymer and Carbon Materials, Polish Academy of Sciences, M. Curie-Sklodowska Str. 34, 41-819 Zabrze, Poland; mgodzierz@cmpw-pan.pl (M.G.); pchaber@cmpw-pan.pl (P.C.); wsikorska@cmpw-pan.pl (W.S.); akobyliukh@cmpw-pan.pl (A.K.); uszeluga@cmpw-pan.pl (U.S.); 2 E.O. Paton Electric Welding Institute of the National Academy of Sciences of Ukraine, 11. Kazymyr Malevych St., 03680 Kyiv, Ukraine; alina.vashchuk@i.ua (A.V.); 4chewip@gmail.com (M.I.); dvaleriyl@ukr.net (V.D.); 3 International Polish-Ukrainian Research Laboratory ADPOLCOM, 41-800 Zabrze, Poland; 4 Laboratory of Modification Polymers, V.P. Kukhar Institute of Bioorganic Chemistry and Petrochemistry of NAS of Ukraine, 50, Kharkivskie Schose, 02160 Kyiv, Ukraine; sergey.rogalsky@gmail.com

**Keywords:** biopolymers, poly(3-hydroxybutyrate), N,N-dibutylundecenoylamide plasticizer, ultrasonic welded joints

## Abstract

This study developed a technical task associated with the formation of welded joints based on biodegradable polymers and their subsequent physicochemical characterization. The primary objective was to establish the effect of the welding process and modification of natural poly(3-hydroxybutyrate) (PHB) with N,N-dibutylundecenoylamide (DBUA) as a plasticizing agent on the structure and properties of PHB-based biopolymer materials as well as the process and structure of welded joints formation using ultrasonic welding technique. The weldability of biodegradable layers based on PHB and PHB/DBUA mixture was ultrasonically welded and optimized using a standard Branson press-type installation. The effect of the DBUA plasticizer and welding process on the structure of PHB-based biodegradable material was investigated using scanning electron microscopy, X-ray diffraction, FT-IR spectroscopy, differential scanning calorimetry, and thermomechanical analysis. The results confirmed that the DBUA acted as an effective plasticizer of PHB, contributing to lower crystallinity of the PHB/DBUA mixture (63%) in relation to the crystallinity degree of pure PHB film (69%). Ultrasonic welding resulted in an additional increase (approximately 8.5%) in the degree of crystallinity in the PHB/DBUA in relation to the initial PHB/DBUA mixture. The significant shift toward lower temperatures of the crystallization and melting peaks of PHB modified with DBUA were observed using DSC concerning pure PHB. The melt crystallization process of PHB was affected by welding treatment, and a shift toward higher temperature was observed compared with the unwelded PHB/DBUA sample. The butt-welded joints of biodegradable PHB/DBUA materials made using the ultrasonic method tested for tensile strength have damaged the area immediately outside the joining surface.

## 1. Introduction

There is an urgent need to replace polymer products of synthetic or petrochemical origin with natural products or those that undergo a relatively rapid biodegradation process [1]. Biopolymers, polymers that are bio-based and/or biodegradable, are suitable materials that can be used to address this need.

Biopolymers can be obtained from various sources, including animals, plants, microorganisms, chemical synthesis of natural origin, and chemical synthesis of fossil sources. Therefore, similar to synthetic plastics, biopolymers can potentially be used in packaging, medicine, and construction, among others [2]. Poly(3-hydroxybutyrate) and its copolymers are isotactic and semi-crystalline that have attracted much attention since their mechanical properties are similar to those of conventional fossil-based thermoplastics such as polypropylene [3,4]. However, PHB has some disadvantages that limit its applications, such as hardness and brittleness due to its high stereoregularity, high degree of crystallinity, and the formation of large and overlapping spherulites with a high tendency to crack [5]. The absence of impurities, acting as a crystallization nucleator, and the stereoregularity of PHB result in a higher nucleation rate when crystallized from the melt. Thermal stability is another drawback that hinders PHB utility. PHB has a narrow processing window and is susceptible to thermal degradation at temperatures close to its melting point, which leads to a degree of degradation during processing and has a negative effect on the mechanical properties [6,7]. This can be resolved by adding low-molecular-weight compounds to the PHB that act as environmentally friendly plasticizers. Fundamentally, the application of plasticizers contributes to the reduction of the glass transition temperature (*T*_g_) and melting temperature (*T*_m_), facilitating thermal processing and reducing the thermal degradation degree. Additionally, these additives improve the toughness and flexibility of the polymer structure by increasing network spacing in the modified PHB material [8]. However, PHB and its copolymers usually have low miscibility with plasticizers. Understanding the separation of the plasticizer between the elastic and rigid amorphous phases and how this affects the thermal, mechanical, and morphological properties remains challenging [9]. The most effective plasticizers are those with low molecular weight and groups available for interaction with the polymer matrix [10]. These plasticizers present good miscibility in the PHB matrix accompanied by a reduction in *T*_g_, an increase in impact strength, and maximum elongation at break. The significant disadvantages of the plasticization procedure include the bigger mass loss at the processing’s temperature range [11] and the migration of plasticizers applied to the PHB surface, causing a noticeable deterioration of key properties of the final material. A large spectrum of various plasticizers has been used with this kind of biopolymers, especially low molecular weight modifying agents, such as citrates, plant oils, esters, glycerol, and oligomeric plasticizers with a molar mass between 1000–6000 g/mol [12], for example, polyethylene-propylene glycol, polyisobutylene, and aliphatic polyesters. Especially, oligomeric plasticizers, such as aliphatic polyester oligomers [13,14], are promising because they are more thermally stable and show less ability to migrate to the PHB surface as a result of lower chain mobility. The presence of a carbonyl group in a plasticizer molecule is important for good miscibility with PHB and for the plasticizing effect. Some tertiary amides of fatty acids, including undecylenic acid amides, have low-temperature plasticizing effects on polyvinyl chloride, comparable to those of commonly used esters. An important advantage of fatty amides over ester-based plasticizers is their antimicrobial activity against bacteria and/or fungi. Thus, tertiary fatty amides are promising bifunctional additives for packaging films since they can play the roles of both plasticizers and antimicrobial agents [15]. However, the plasticizing effects of these compounds on PHB have not been studied.

Recently, innovative research on the applications of biopolymers, including PHB-based systems, for welding technologies has been conducted to address the needs of chemical, food, and other industries for the manufacture of film containers and packaging [16]. The most popular welding methods include hot tool welding, resistance welding, ultrasonic welding, and high-frequency welding. Butt welding and lap welding are also universal methods that can be applied to polymeric materials. Among the methods of plastic welding, the ultrasonic technique is an ultrafast and energy-efficient solid-state bonding process that functions on the principle of the application of high frequencies (typically between 20 and 50 kHz) and low amplitude vibrations (typically between 10 and 250 μm) at the interface of the joining surfaces of the adherents to be welded [17,18]. Moreover, the ultrasonic welding of plastics is an eco-friendly process with low energy consumption while producing a cost-effective, permanent, and clean welding seam. Therefore, this technique is widely used for packaging applications. Welding is considered the most effective method for thermoplastic polymeric materials such as PLA and PHB. Klinstein et al. [19] have reported the weldability of PLA using two different methods, namely impulse high frequency and ultrasonic welding. Also, the authors investigated the impact of the weld joints on the mechanical properties of the welded material and showed that in the case of impulse welding, excessive weld energy and long heating time with high current values lead to film damage and low weld strengths. In the case of ultrasonic welding, the weld strength is proportional to the weld distance and inversely proportional to the weld/melt velocity. The small changes in the thermal properties of PLA and PHA samples in the region of the weld were only observed when the ultrasonic weld was used [18]. Pagano et al. have demonstrated that it is possible to join PLA to aluminum thin films for food-packaging applications using laser transmission welding. The joint quality depends on the power level of the laser source [20].

In this study, N,N-dibutylundecenoylamide (DBUA) was assessed as a potential eco-friendly plasticizer for PHB-based films, and the plasticizing effect of DBUA on the formation of modified PHB/DBUA films and their structure and thermal characteristic as well as on the welding process was investigated. The thermal phase transitions, such as crystallization and melting, and the crystallinity degree were determined using DSC. The results were analyzed in conjunction with XRD studies and morphological data of the systems, making it possible to evaluate the effects of plasticizer and welding process on the structure and thermal properties of biopolymer welding joints. Finally, thermomechanical analysis of plasticized PHB samples was performed to determine the dimension changes as a function of temperature, thermal expansion coefficient, and glass transition temperature.

## 2. Materials and Methods

### 2.1. Materials

The following chemicals were obtained from Sigma-Aldrich (St. Louis, MI, USA): undecenoic acid (98%), dibutylamine (99.5%), thionyl chloride (97%), chloroform (99%), and dichloromethane (99.8%). Suspension polyvinyl chloride KSF-70 was purchased from Karpatnaftochim (Ukraine).

The commercially available poly(3-hydroxybutyrate), the ENMAT Y1000 brand (Tianan Biologic Material Co., Ningbo, China), a semi-crystalline thermoplastic polymer with a density of ρ = 1.25 g/cm^3^ and a melting temperature *T*_m_ = 170–176 °C was used as a biodegradable component of thin films and welded joints. The raw PHB polymer was dissolved in chloroform, precipitated in hexane, and dried in a vacuum at a temperature of 40 °C [5,14]. N,N-dibutylundecenoylamide (Table 1) was synthesized and purified as previously described [20,21], and their purity was confirmed using ^1^H NMR spectroscopy.

^1^H NMR (400 MHz, CDCl_3_) δ: 0.93 (t, 6H, CH_3_), 1.3 (m, 14H, (CH_2_)_5_, -NCH_2_CH_2_CH_2_), 1.49 (m, 4H, NCH_2_CH_2_), 1.63 (m, 2H, -CH_2_CH_2_CO-), 2.03 (m, 2H, -CH_2_=CH-CH_2_-), 2.28 (t, 2H, -CH_2_CO-), 3.2 (t, 2H, NCH_2_), 3.3 (t, 2H, NCH_2_), 4.94 (m, 2H, -CH_2_=CH-), 5.82 (m, 1H, -CH_2_=CH-).

### 2.2. Preparation of Plasticized PHB Films

The film formation process was experimentally optimized to obtain the most amorphous structure possible with a low concentration of plasticizer. PHB solutions in chloroform were prepared at a concentration of 10 g/100 mL. To this solution, 10 wt.% DBUA was added. The mixtures were poured onto a Teflon support, and the solvent was allowed to evaporate at room temperature for 24 h. Residual solvent was removed in a 10-mbar vacuum at 60 °C for 12 h. To obtain good quality PHB/DBUA films, samples were placed in a press mold with subsequent heating; the temperature of the mold should be set in the range of PHB melting temperatures (i.e., between 170 °C and 180 °C), and the minimum heating time should be 45 min. It was established that for the formation of high-quality films based on PHB, the temperature of the mold should be set in the range of melting temperatures of PHB, i.e., between 170 °C and 180 °C, and the heating time should be approximately 45 min. Using these parameters, it was found that high-quality films were formed with a small capacity for thermal degradation. The higher the temperature above 180 °C, the higher the capacity of the films for thermal degradation. The thickness of polymer films was approximately 150 μm.

### 2.3. Welding

PHB films were squeezed together at room temperature for welding on a Branson 8400 model of press-type installation (Branson Co., CO, USA). A sonotrode with a flat horizontal surface of the two types was used—a smooth polished surface and a profiled surface with regular pyramidal protrusions of the “knurl” type. The sonotrode was driven by an electrical signal of 20 kHz, which resulted in ultrasonic mechanical vibrations with an amplitude of 40–50 μm. The welding time (5–7 s) and pressure (0.3 MPa) were held constant.

**Figure 1:**
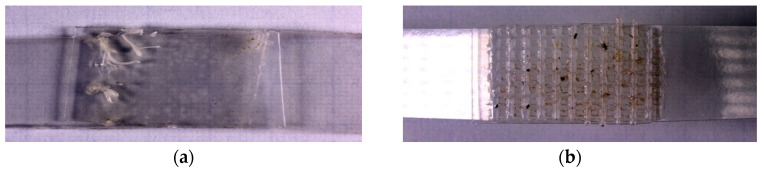
Digital images of ultrasonic welded joints based on the PHB/DBUA biodegradable film made by the centroid with a flat (**a**) and profiled (**b**) surface.

The externally visible defects of the surface of the welding seams were tested by visual inspection (Figure 1).

The butt welds jointed with the sonotrode with the flat surface formed well, but the heating of the material was somewhat uneven across the joint area (Figure 1a). Fillet seams, welded with a profiled sonotrode, were formed with the formation of uniform penetration over the entire joint area (Figure 1b).

### 2.4. Characterization Methods

Microscopic studies were performed with scanning electron microscopy (SEM) using a scanning electron microscope Quanta 250 FEG (FEI Company, Hillsboro, OR, USA) operating at an acceleration voltage of 10 kV. Samples were analyzed uncoated under low vacuum (80 Pa). XRD studies were performed on PHB-based samples using the D8 Advance diffractometer (Bruker AXS, Karlsruhe, Germany) with Cu-Kα cathode (λ = 1.54 Å) operating at 40 kV voltage and 40 mA current. The scan rate was 0.60°/min with scanning step 0.02° in the range of 2° to 70° 2Θ. Identification of fitted phases was performed using DIFFRAC.EVA program with the use of ICDD PDF#4 database. Lattice parameters of the fitted phase were calculated using Rietveld refinement in the TOPAS 6 program, based on Williamson-Hall theory [22,23]. Rietveld refinement allows for the calculation of crystallite size and lattice strain of the crystalline part of PHB [24,25], while the crystallinity of the polymer was calculated using a peak decomposition method [26]. For the structural analysis, the unit cell parameter a is related to the short polymer axis, and c corresponds to the long axis of the molecule, while b is related to the inter-chain interactions.

Fourier transform infrared (FTIR) spectra were collected using a JASCO FT/IR-6700 (JASCO Co., Ltd., Tokyo, Japan) spectrometer, which was equipped with a single reflection ATR accessory having a diamond crystal (ATR PRO670H-S). The infrared spectra were measured with a resolution of 4 cm^−1^ over a spectral range of 400–4000 cm^−1^. A total of 32 scans were accumulated for each sample. The ATR-FTIR measurements were carried out directly on the PHB films with no sample pretreatment.

Thermal analysis was performed using a DSC Q2000 (TA Instruments Waters Co., New Castle, DE, USA) differential scanning calorimeter under a nitrogen atmosphere with a nitrogen flow rate of 50 mL/min to eliminate oxidative degradation, to which the PHB is susceptible [27]. The baseline calibration in the temperature ranges from −100 to 300 °C, and the calibration with the indium standard was performed. Samples for tests of approximately 10 mg were encapsulated in standard non-hermetic aluminum. The following four thermal cycles were used: (1) heating from −30 °C to 200 °C (first heating run); (2) isothermal treatment: the samples were held at 200 °C for 5 min to eliminate a residual crystallinity and remove the previous thermal history; (3) the melt material was cooled to −30 °C (cooling run) and (4) reheated to 200 °C (second heating run). Tests were conducted at constant heating and cooling rate of 10 °C/min.

The melting temperature (TM) and the melting enthalpy (heat of fusion, Δ*H_m_*) were de-terminated in accordance with ISO 11357-3 standard. The degree of crystallinity (Xc (%)) for pure, modified with DBUA, and welded PHB/DBUA samples were calculated as follows:

$$X_{c}\left(\%\right)=\frac{\Delta H_{m}}{\Delta H_{0}\times w_{PHB}}\times 100\%$$

where Δ*H_m_* is the experimental melting enthalpy value of the sample (J/g) obtained in the second heating DSC cycle, Δ*H*_0*m*_ is the melting enthalpy of 100% perfectly crystalline form of PHB (146 J/g) [24,27,28], and *w_PHB_* is the weight fraction of PHB in the plasticized sample.

Thermomechanical studies of polymer systems were performed using thermomechanical analyzer Discovery 450 TMA (TA Instruments, Waters Co., USA) with the penetration method in the mode of uniaxial constant load (σ = 0.5 MPa) using the UIP-70M unit. Linear heating of the samples was performed at a rate of 2.5 °C/min [29,30]. The research was conducted in the temperature range from 20 to 200 °C.

## 3. Results

**Figure 2:**
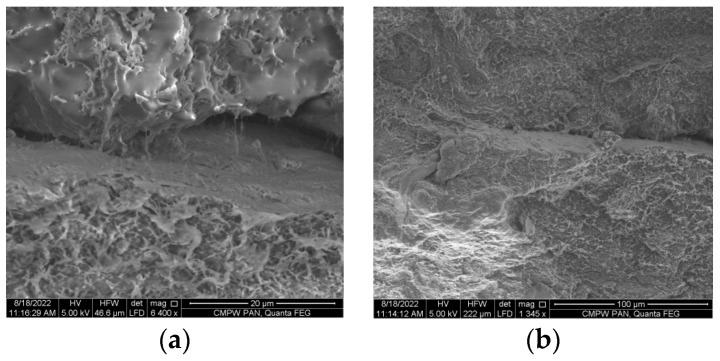
SEM images of the PHB/DBUA biopolymer film out-of-weld area (**a**) and PHB/DBUA biopolymer welded area (**b**).

The SEM results show the homogeneity of the structure of the initial PHB/DBUA material studied on the surface outside the weld and the surface of the welded joint (Figure 2). There is no visible separation process and melt zones, which confirms the uniform structure of the weld formation (Figure 2b).

The SEM microphotographs show the porous structure of the PHB/DBUA film, which indicates the low density of the material and its plasticity. In the microphoto of the weld seam, a melted zone is visible, which indicates the quality of welding processes and good weld cohesion of the weld.

**Figure 3:**
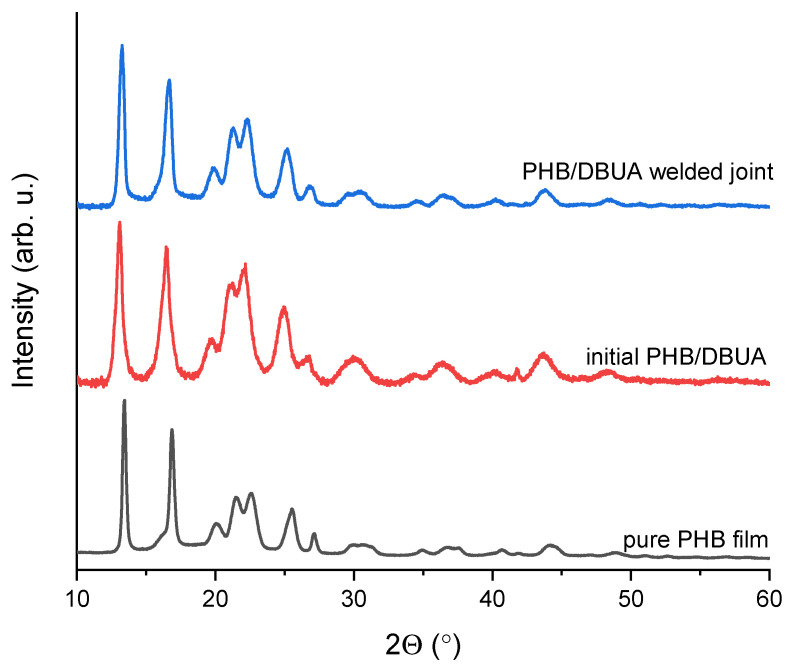
XRD patterns of pure PHB film and initial PHB/DBUA and PHB/DBUA welded joint.

The XRD studies of the original PHB are shown to evaluate the effect of its modification by DBUA. Figure 3 presents XRD patterns for the initial PHB film, welded joint made of PHB modified with DBUA, and for the PHB/DBUA area out-of-weld. All peaks observed on registered patterns correspond to a crystalline phase of PHB with P212121 orthorhombic space group (PDF#00-068-1411).

The strong decrease of (020) and (110) peaks observed (two first ones), with no changes in (101) and (111) peaks (around 22° 2Θ), results in lower crystallinity of the initial PHB/DBUA (64.1%) and PHB/DBUA welded joint area (67.5%) in comparison with pure PHB film (70.0%).

Table 2 presents selected structural parameters determined by Rietveld refinement. Based on them, we concluded that ten times lower crystallite size and four times lower lattice strain were found for the initial PHB/DBUA as compared with PHB/DBUA welded joint area, possibly due to plasticizer addition or ultrasound treatment affecting the molecular arrangement of PHB chains.

In particular, ultrasound treatment can increase the temperature during welding, contributing to partially melting the PHB crystals and their crystallization during cooling, resulting in higher crystallite size and crystallinity in the welded area compared to the initial PHB/DBUA. These phenomena can be confirmed by calculating the lattice parameters (Table 2), especially by the significant increase of the c parameter in the welded area compared to the out-of-weld area. It can be noted that the PHB crystal sizes reflect a slight reduction in the short polymer chain alignment and inter-chain interactions in comparison to model data from the ICDD PDF#4 database, however, these structural parameters are directly related to the fabrication method.

**Figure 4:**
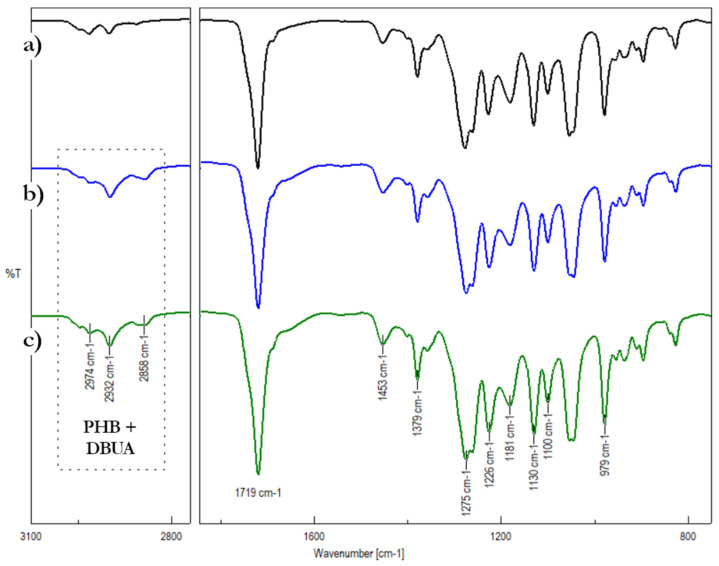
ATR-FTIR spectra in two regions 1850 and 750 cm^−1^ (right side and 3100 and 2750 cm^−1^ (left side of the picture) for the samples: (**a**) PHB in powder form (**b**) unwelded PHB/DBUA packaging film and (**c**) welded PHB/DBUA packaging film.

Attenuated total reflectance-Fourier transform infrared (ATR-FTIR) spectroscopy has provided information about the vibrational motions of molecules located within 1–2 μm of the surface layer of a sample 31. The ATR-FTIR spectra of the PHB powder and the out-of-weld and welded area of the PHB film plasticized with DBUA are shown in Figure 4.

All spectra display characteristic absorption bands of the PHB homopolymer. A strong band at 1719 cm^−1^ corresponds to the C=O stretching vibration of the PHB, while the vibrational bands related to the ester C–O–C bond appear at 1275, 1181, and 1100 cm^−1^ [31]. The FTIR peaks occurring in the 2800–3050 cm^−1^ range are due to CH_2_ and CH_3_ stretching vibrations of PHB and DBUA plasticizer [32,33]. The absorption band at 1130 cm^−1^ may be assigned to the CH_3_ rocking vibrations [32]. FTIR spectroscopy makes it possible to distinguish bond vibrations for a polymer originating from the crystalline phase from those originating from the amorphous state and to compare the crystallinity degree for differentially prepared samples [15].

**Figure 5:**
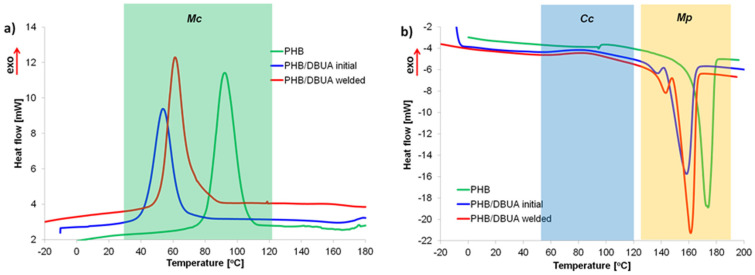
Crystallization exotherms (second run) (**a**) and melting endotherms preceded by cold crystallization exotherms (third run) (**b**) of PHB and PHB/DBUA samples.

The intensity ratio between the bands at 1226 and 1453 cm^−1^ is most frequently used to evaluate the crystallinity of PHB samples [34,35,36], and the intensity of the band at 1226 cm^−1^ (the C–O–C stretching vibration of PHB) correlates well with the crystalline content of the homopolymer [37,38]. The band at 1453 cm^−1^ is, in turn, insensitive to the physical state of PHB. The I1226:I1453 intensity ratio for out-of-weld area is 4.12. In the case of the welded area of the plasticized PHB film, this ratio is 4.51, which confirms that the welded PHB/DBUA specimen exhibits higher crystallinity than the initial PHB/DBUA mixture. This is consistent with the XRD and DSC results, indicating that the crystallinity of PHB increased due to the welding process. The effect of PHB modification using DBUA as a plasticizing agent, as well as the welding process on crystallization, cold crystallization, and melting parameters of PHB/DBUA systems was studied using the DSC technique. Figure 5 illustrates the crystallization (a) and melting during third DSC runs (b) characteristic of PHB-based materials.

The detailed melt crystallization peak maximum (*T*_Mc_) and enthalpy (Δ*H*_Mc_), cold crystallization peak temperature (*T*_Cc_) and enthalpy (Δ*H*_Cc_), and melting peak temperature (*T*_m_) with enthalpy value (Δ*H*_m_) are summarized in Table 3. The *X*_c_ is the calculated crystallinity degree of PHB.

The curve of the third DSC run related to the melting of pure PHB film (Figure 5b) shows the endothermic peak with a slight shoulder at a lower temperature before the main crystal melting temperature at ~174 °C. Double or more complex melting behaviors of PHB have been described and related to the partial melting, remelting, recrystallization, or melting of crystals of different structures (β crystals melting at a lower temperature than the most frequently occurred α form of PHB), perfection, or thickness [39]. This additional shoulder can be associated with the smaller and more imperfect crystals formed in relation to the main melting peak at a higher temperature of more perfect (recrystallized) crystals.

Additionally, the cold crystallization of PHB was noticed with a blurred maximum at approximately 100 °C (Figure 5b). The cold crystallization was not observed during the first heating run of the fully crystalline phase of PHB as obtained after processing and a period of storage, whereas the second repeated heating refers to the melting of PHB obtained during the more controlled cooling stage of the experiment [38,40]. A melt crystallization event for the PHB crystalline phase was recorded during cooling after the first heating run. The crystallization peak at 92 °C with enthalpy of crystallization of 83 J/g were observed, consistent with previous findings [41]. The crystallinity degree of pure PHB film was 69%, higher than PHB modified with DBUA by approximately 10.5%.

As shown in Figure 5, the welding process significantly influenced all phase transition events. PHB partially crystallizes from the melt during the cooling step and partially cold crystallized during the reheating DSC step. Observing melt crystallization (Figure 5a) and cold crystallization (Figure 5b), we concluded that these phenomena are affected by welding treatment. Melt crystallization for the PHB/DBUA sample taken from the welded area was promoted with higher *T*_Mc_ (61 °C) compared to the sample not subjected to welding with *T*_Mc_ (54 °C). During cooling, a downshift of the crystallization peak temperature was induced by the presence of the DBUA plasticizer the PHB. The crystallization enthalpy of 49.2 J/g after welding was higher than the PHB/DBUA out-of-weld area. Also, cold crystallization parameters (i.e., Δ*H*_Cc_ and *T*_Cc_) were higher for the sample after welding than for the initial PHB/DBUA material before welding treatment.

During the second heating run, for the melting event, all samples showed double endothermic peaks. An initial noticeable smaller melting peak was followed by a larger peak at a higher temperature. The melting temperature peak of neat PHB was 173 °C [29]. The addition of DBUA plasticizer resulted in a slight decrease in the *T*_m_ of the PHB; consequently, the processing temperature window became wider making the plasticized PHB mixtures easier to process. The melting enthalpy of PHB was also reduced with the plasticizer addition. The small peak was more pronounced and shifted toward a higher temperature when the welding process was applied to the PHB/DBUA mixture.

Double peaks appearing in the melting region may result from the presence of more crystalline varieties (polymorphism), i.e., melting of crystals of different structures (β crystals melting at a lower temperature than the most frequently occurred α form of PHB), different type of morphology (lamellae thickness, distribution, stability), or polymer phase of various molecular weight [42,43,44]. The basic melting peak of PHB/DBUA after the welding was shifted approximately 3 °C toward a higher temperature and was characterized by higher total melting enthalpy as compared with the initial sample. The low melting temperature of PHB/DBUA films is related to the original melt-crystallized lamellar structure. In turn, the higher melting peak temperature for PHB modified with DBUA after the welding procedure can result from structural reorganization into thicker and more stable PHB crystals.

This can be estimated using the Gibbs-Thompson equation representing the relationship between the melting temperature of the partially crystalline polymers determined during the third DSC run and the lamella thickness (*L*) as follows [44,45]:

$$L=\frac{2\sigma_{e}T_{m}^{0}}{\Delta H_{f}\left(T_{m}^{0}-T_{m}\right)}$$

where $T_{m}^{0}$ is the equilibrium melting point with a best-fit value of 197 ± 2 °C [46,47], Δ*H_f_* is the melting enthalpy, and *σ*_e_ is the free energy of formation of the lamellae surface, it is constant, *σ_e_*/Δ*H_f_* is equal for PHB 2.06 × 10^−10^ m^−1^ [48]. The primary two assumptions were (1) the two other crystal dimensions are large in relation to *L* and (2) the heat capacity for liquid and solid form PHB are approximately equal in the melting region.

The lamellae thickness of the crystallites calculated based on the melting peak temperature for the initial pure PHB film is 96.4 Å. The influence of the welding process on the lamellar thickness of the crystallites is shown in Table 3. For the out-of-weld area of PHB film, *Lc* is 10.1 and 25.1 Å. The welding process promotes the growth of crystalline zones with higher lamellae sizes. The heterogeneity of the material increases as a result of the welding treatment. The formation of short chains causes the “cold” crystallization enthalpy to increase.

As shown in Table 3, there was an 8.5% increase in the degree of crystallinity in the PHB/DBUA welded sample in relation to the initial PHB/DBUA. It stands with good agreement calculated via the XRD and FTIR methods. The addition of a plasticizer may affect the course of the PHB crystallization process in various ways. It can both reduce and increase the degree of PHB’s crystallinity due to dilution. The observed increase for composites studied can be related to the decrease in the melt viscosity, which results in higher chain diffusion and a faster crystallization rate [16,49]. The slight differences between crystallinity degrees determined using the XRD and DSC techniques result from measurement conditions. DSC thermogram represents phase transitions as a function of temperature, but it should be noted that the peak maximum of the melting DSC endotherm does not represent the melting temperature of the original crystals. Upon heating, the crystals are constantly melting and structurally reorganizing. The melting peak temperature is the largest difference between melting and recrystallization processes and strongly depends on the DSC heating conditions.

**Figure 6:**
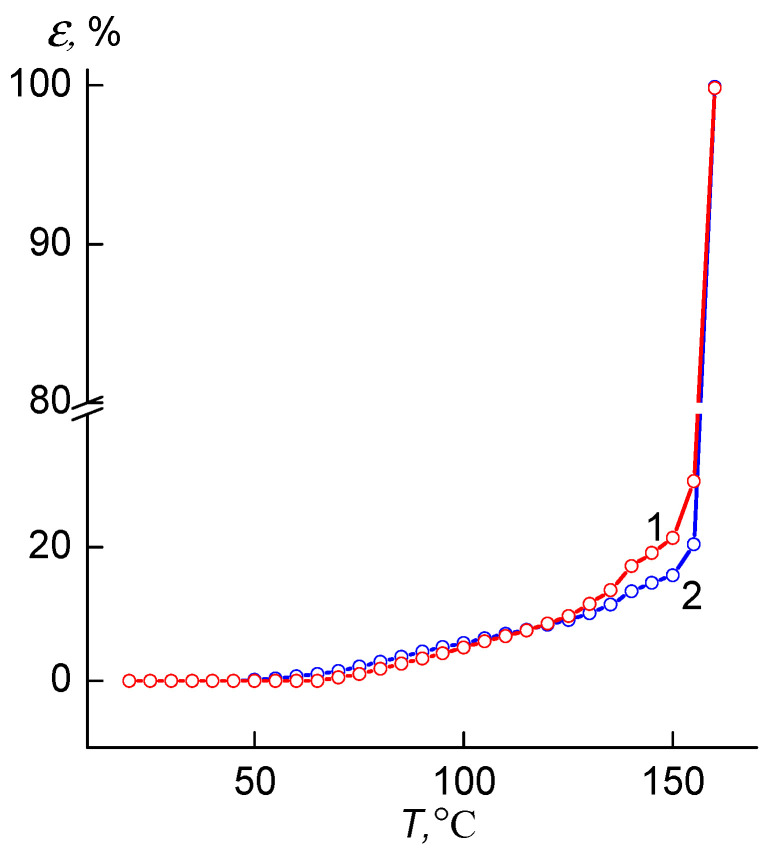
Thermomechanical curves of the original PHB/DBUA film (1) and the welded joint based on PHB/DBUA mixture (2).

The analysis of thermomechanical curves of the initial PHB/DBUA and the welded joint based on it showed (Figure 6) that in the temperature range of 70 °C (curve 1) and 60 °C (curve 2), there are temperature transitions associated with the onset of temperature mechanical vitrification of these samples. It should be noted that of the welded joint, the temperature of the transition to the viscous state is 155 °C, slightly higher than that of the original PHB/DBUA (*T* = 153 °C). The relative deformation of the initial sample of PHB/DBUA and the welded joint based on it is insignificant and amounts to 5–6% (at *T* = 100 °C).

**Figure 7:**
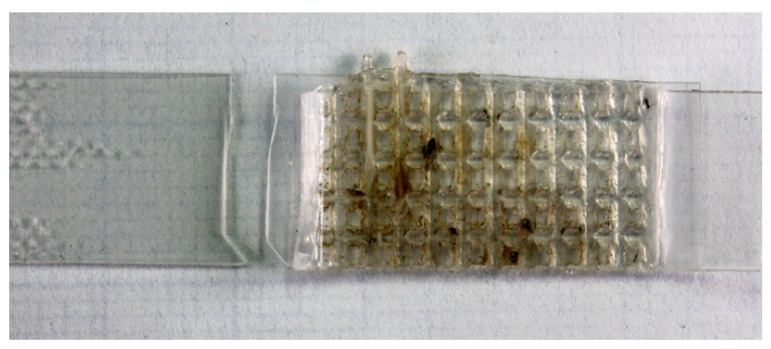
Destruction of the cover weld during mechanical tests.

After the welding processes, a study was conducted on the tensile strength of the welded joints. All butt-welded joints of the biodegradable films made using the ultrasonic method were destroyed along the main material out of the welded joint during mechanical uniaxial tensile tests (Figure 7) due to the concentration of mechanical stresses at the end of the profiled surface of weld areas.

## 4. Conclusions

The experimental work revealed that biopolymer samples based on commercial polyhydroxybutyrate modified with 10 wt.% of N,N-dibutylundecenoylamide plasticizer can create butt welds jointed using ultrasonic welding. These welds were characterized by a flat surface with small fragments of destroyed polymer material due to the concentration of mechanical stresses and the temperature rise on the profiled surface of the sonotrode, contrary to the typical thermal welding procedures. However, due to these local stresses at joining points, all butt-welded joints of biodegradable films made using the ultrasonic method were destroyed along the main material out of the welded area during mechanical uniaxial tensile tests.

The DBUA acted as a plasticizer of PHB, and a decrease in the crystallinity of PHB/DBUA mixture determined using the DSC technique to the level of approximately 63% was observed as compared with the crystallinity degree of pure PHB film equal to approximately 69%. The ultrasonic welding resulted in an 8.5% increase in the degree of crystallinity in the PHB/DBUA in relation to the initial PHB/DBUA mixture. This stands in good agreement with values calculated via XRD and FTIR methods.

Chemical transformations that do not occur in biodegradable PHB/DBUA films formed by dissolution or pressing, but as a result of the welding process, changes in the phase structure of biopolymers have been revealed.

The significant shift toward lower temperatures of the crystallization peak of PHB modified with DBUA was observed by DSC concerning pure PHB. The melt and cold crystallization processes of PHB were affected by welding treatment. During cooling, a downshift of crystallization peak temperature was induced by the presence of DBUA plasticizer PHB. Melt crystallization for the PHB/DBUA sample taken from the welded area is promoted in relation to the sample not subjected to welding. Similarly, cold crystallization peak temperature and enthalpy were higher for the sample after welding than for the initial PHB/DBUA material before welding.

The behavior of PHB was also affected by the addition of DBUA plasticizer and welding. Adding a DBUA plasticizer resulted in a slight reduction in the PHB melting temperature and led to the broadening of the processing temperature window, making the plasticized PHB mixtures easier to process.

Future studies on the effect of the DBUA plasticizer and the ultrasonic welding process on the PHB biodegradation process are planned. Future research that includes graphene derivatives as electroactive components, especially reduced graphene oxide forms with the natural ability to capture radicals providing packaging materials with an antioxidant effect, and the characteristics of PHB-based systems will help to reduce their present limitations on mechanical strength as well as thermal and electrical parameters.

## Figures and Tables

**Table 1:** Chemical structure and basic physico-chemical characteristics of components.

Component	Structure	Characteristics
Polyhydroxybutyrate (PHB)	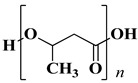	d = 1.25 g/cm^3^ *T*_melt_ = 170–176 °C
N,N-dibutylundecenoylamide (DBUA)	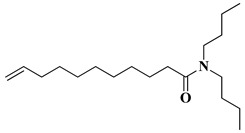	*T*_Δm = 5%_ = 219 °C

*T*_melt—_melting temperature; *T*_Δm = 5%_ temperature at which a 5% mass loss is observed during thermogravimetric analysis.

**Table 2:** Structural parameters of PHB crystalline phase determined by Rietveld refinement.

	Model Parameters, ICDD, Card No. 00-068-1411	Pure PHB Film	Initial PHB/DBUA Film	PHB/DBUA Welded Joint
Lattice parameters, Å	a = 5.73,	a = 5.682,	a = 5.679,	a = 5.678,
	b = 13.098,	b = 13.046,	b = 13.058,	b = 13.063,
	c = 5.958	c = 5.946	c = 5.95	c = 5.968
Crystallite size, nm	-	89 ± 10	9.7 ± 0.3	100 ± 26
Lattice strain, %	-	2.98 ± 0.04	0.84 ± 0.22	3.55 ± 0.08

**Table 3:** Crystallization and melting parameters of PHB-based systems determined using DSC.

Biopolymer System	Crystallization from Melt		Cold Crystallization		Melting					*X*_c_ [%]
	*T*_Mc_ [°C]	Δ*H*_Mc_ J/g]	*T_C_*_c_ [°C]	Δ*H_C_*_c_ [J/g]	*T*_m1_ [°C]	*L* [Å]	*T*_m2_ [°C]	*L* [Å]	Δ*H*_m_ [J/g]	
Pure PHB	92.2	82.9	99.8	8.8	-	-	173.7	96.4	101.2	69.4
PHB/DBUA initial	53.6	43.9	85.8	11.3	136.8	10.1	158.4	25.1	64.2	62.8
PHB/DBUA welded	61.1	49.2	86.8	13.4	143.4	18.1	161.3	27.1	69.6	68.1

## Data Availability

Not applicable.
